# EGFR phosphorylates and inhibits lung tumor suppressor GPRC5A in lung cancer

**DOI:** 10.1186/1476-4598-13-233

**Published:** 2014-10-14

**Authors:** Xiaofeng Lin, Shuangshuang Zhong, Xiaofeng Ye, Yueling Liao, Feng Yao, Xiaohua Yang, Beibei Sun, Jie Zhang, Qi Li, Yong Gao, Yifan Wang, Jingyi Liu, Baohui Han, Y Eugene Chin, Binhua P Zhou, Jiong Deng

**Affiliations:** Department of Pathophysiology, Key laboratory of Cell Differentiation and Apoptosis of Minister of Education, Shanghai Jiao Tong University School of Medicine, Shanghai, 200025 China; Shanghai Key Laboratory for Tumor Microenvironment and Inflammation, Shanghai Jiao Tong University School of Medicine, Shanghai, 200025 China; Department of Thoracic Surgery, Shanghai Chest Hospital, Shanghai Jiao Tong University, Shanghai, 200030 China; Translation Medicine Center, Shanghai Chest Hospital, Shanghai Jiao Tong University, Shanghai, 200030 China; Department of Pathology, Shanghai Chest Hospital, Shanghai Jiao Tong University, Shanghai, 200030 China; Department of Oncology, Shanghai First People’s Hospital, Shanghai Jiao Tong University School of Medicine, Shanghai, 200120 China; Department of Oncology, Shanghai East Hospital, Tongji University, Shanghai, 200120 China; Department of Molecular and Cellular Biochemistry, Markey Cancer Center, University of Kentucky College of Medicine, Lexington, KY 40506 USA; Insitute of Health Science, Shanghai Institute of Biological Science, Chinese Academy of Science, Shanghai, 200025 China; Biopeptek, Inc, Malvern, PA 19355 USA; Gallus Biopharmaceuticals NJ, LLC, Princeton, New Jersey 08540 USA

**Keywords:** GPRC5A, EGFR, Tyrosine kinase, Lung cancer, Post-translation modification

## Abstract

**Background:**

GPRC5A is a retinoic acid inducible gene that is preferentially expressed in lung tissue. *Gprc5a*– knockout mice develop spontaneous lung cancer, indicating Gprc5a is a lung tumor suppressor gene. GPRC5A expression is frequently suppressed in majority of non-small cell lung cancers (NSCLCs), however, elevated GPRC5A is still observed in a small portion of NSCLC cell lines and tumors, suggesting that the tumor suppressive function of GPRC5A is inhibited in these tumors by an unknown mechanism.

**Methods:**

In this study, we examined EGF receptor (EGFR)-mediated interaction and tyrosine phosphorylation of GPRC5A by immunoprecipitation (IP)-Westernblot. Tyrosine phosphorylation of GPRC5A by EGFR was systematically identified by site-directed mutagenesis. Cell proliferation, migration, and anchorage-independent growth of NSCLC cell lines stably transfected with wild-type GPRC5A and mutants defective in tyrosine phosphorylation were assayed. Immunohistochemical (IHC) staining analysis with specific antibodies was performed to measure the total and phosphorylated GPRC5A in both normal lung and lung tumor tissues.

**Result:**

We found that EGFR interacted with GPRC5A and phosphorylated it in two conserved double-tyrosine motifs, Y317/Y320 and Y347/ Y350, at the C-terminal tail of GPRC5A. EGF induced phosphorylation of GPRC5A, which disrupted GPRC5A-mediated suppression on anchorage-independent growth of NSCLC cells. On contrary, GPRC5A-4 F, in which the four tyrosine residues have been replaced with phenylalanine, was resistant to EGF-induced phosphorylation and maintained tumor suppressive activities. Importantly, IHC analysis with anti-Y317/Y320-P sites showed that GPRC5A was non-phosphorylated in normal lung tissue whereas it was highly tyrosine-phosphorylated in NSCLC tissues.

**Conclusion:**

GPRC5A can be inactivated by receptor tyrosine kinase via tyrosine phosphorylation. Thus, targeting EGFR can restore the tumor suppressive functions of GPRC5A in lung cancer.

## Background

The retinoic acid inducible G protein-coupled receptor family C group 5 member A (GPRC5A) is expressed predominantly in normal lung tissue [1]. Several lines of evidences suggest that it function as a lung-specific tumor suppressor: a) *Gprc5a*^-/-^ mice develop spontaneous lung tumors [2]; b) loss of heterozygosity of chromosome 12p, where GPRC5A gene (12p12.3-p13) resides, is common in NSCLCs [3, 4]; and c), GPRC5A expression is suppressed in many lung cancer cell lines and tumor tissues compared to adjacent normal lung tissues [2, 5]. In addition, we showed previously that lung tissues from *Gprc5a*^*-/-*^ mouse have increased NF-κB activation compared with that of wild-type mice [6]. This suggests that Gprc5a negatively regulates a subset of genes involved in inflammation, proliferation, and survival. However, there are several controversy reports showing that elevated GPRC5A expression was found in some tumor cell lines and tumor samples and this expression correlated with increased cell growth and colony formation [7–9]. One possible explanation for these opposite observations is that the tumor-suppressive function of GPRC5A can be inactivated under certain conditions. Understanding the underlying mechanisms of GPRC5A functions will yield new insights into lung tumorigenesis and permit development of novel therapeutic invention for restoring the tumor suppressive functions of GPRC5A in lung cancer.

G protein-coupled receptors (GPCRs) can be modified by glycosylation, phosphorylation and palmitoylation, which alter protein conformation, protein association, subcellular localization, and/or biological functions [10, 11]. For example, GPCRs are desensitized via phosphorylation following agonist stimulation. This phosphorylation is directed to serine/threonine residues in the cytoplasmic tail and third cytoplasmic loop but rarely on tyrosine residues. The Ser/Thr phosphorylation by GPCR kinases (GRKs) leads to the internalization of GPCRs [10, 11] and hampers GPCR signaling [12]. GPCRs can also undergo Tyr-phosphorylation on residues located in the cytoplasmic domain [13]. It has been suggested that tyrosine phosphorylation of GPCR is required for Src recruitment and subsequent Ser/Thr phosphorylation by GRK. In some GPCRs, a tyrosine containing motif in the cytoplasmic tail has been linked to the internalization of GPCRs. For example, cytokine-induced tyrosine phosphorylation of CXCR4, which reduces the level of functional CXCR4 on cell surface, contributes to GRK3 and β-arrestin2-mediated sequestration of this receptor in the cytoplasm [14]. It remains elusive whether GPRC5A is subjected to phosphorylation, leading to altered activities in lung cells.

EGFR and its family members are the major groups of receptor tyrosine kinases that are aberrantly activated in many NSCLCs [15]. EGFR is over-expressed in more than 60% of NSCLC cases [16]. In addition, oncogenically-activated mutant forms of EGFR and HER2 have been found in lung cancer [17], and contribute to the development of this disease [18]. Moreover, EGF and TGF-α, ligands of EGFR, are frequently expressed in NSCLCs, which provides an autocrine mechanism to sustain the hyper-activation of these receptor tyrosine kinases (RTKs) [19]. In an un-biased whole cell phospho-peptide analysis, GPRC5A was identified as one of the tyrosine phosphorylated protein in HER2-overexpressing HMEC cells after EGF or heregulin (HRG) treatment [20, 21]. This suggests a potential cross-regulation between EGFR and GPRC5A. In this study, we showed that EGFR interacts with and phosphorylates GPRC5A in two highly conserved double-tyrosine modules (Y317/Y320 and Y347/Y350) at the C-terminal domain of GPRC5A. EGF treatment inhibited GPRC5A-mediated repression of anchorage-independent growth via phosphorylation of these tyrsoine sites since the same treatment failed to do so on GPRC5A-4 F mutant, in which tyrosine residues were replaced with phenylalanine (F). IHC analysis with specific antibody to Y317/Y320-P site showed that GPRC5A in NSCLC tissues is mostly phosphorylated, whereas GPRC5A in adjacent tumor tissues is mostly non-phosphorylated. Thus, EGFR-mediated tyrosine phosphorylation represents a newly identified mechanism by which the tumor suppressive function of GPRC5A is inactivated in lung cancer.

## Results

### EGFR interacts with and phosphorylates GPRC5A

**Figure 1:**
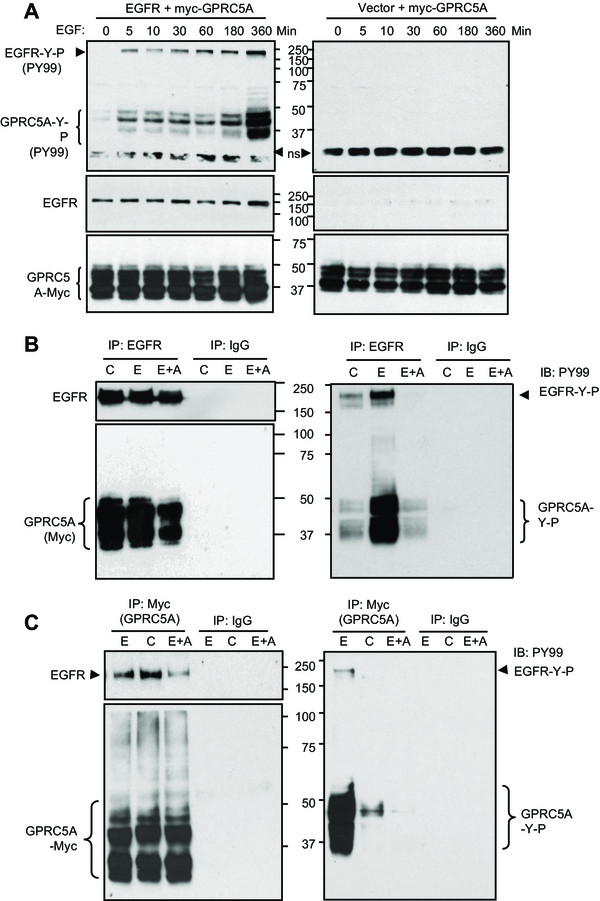
**EGFR complexes with and tyrosine phosphorylates GPRC5A. A**, HEK293T cells were transfected with the plasmids encoding myc-tagged GPRC5A plus either EGFR or empty vector. Cells were treated with EGF (100 ng/ml) for different time periods as indicated. Cell lysates were harvest for Western blot using antibody PY99 (anti-pan-phospho-tyrosine), or anti-EGFR, or anti-myc-tag. **B-C**, Cells were co-transfected and treated as indicated. Treatment groups include: C- Control; E- EGF (100 ng/ml, 5 min); E + A- EGF + AG1478. Cell lysates were harvest for immuno- precipitation (IP)-Western blot analysis with antibodies as indicated.

**Figure 2:**
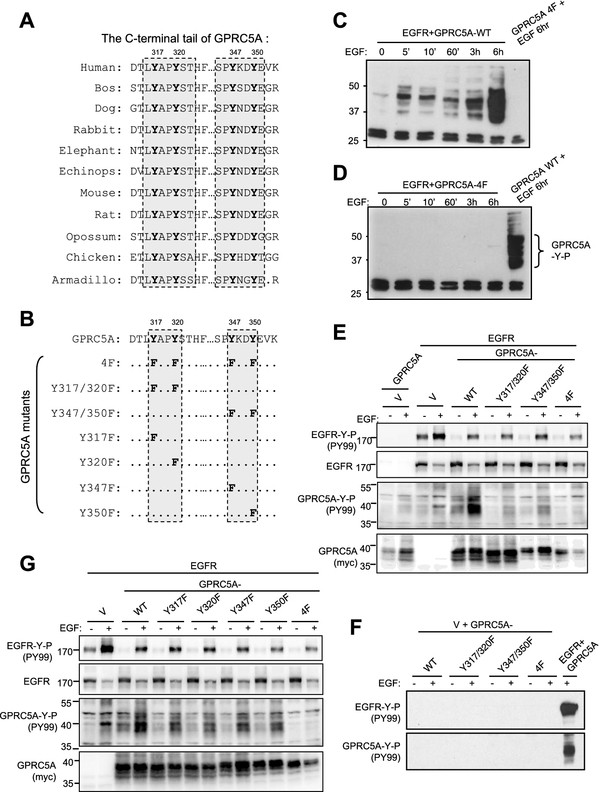
**EGFR mainly phosphorylates the tyrosine residue Y317/Y320 of GPRC5A. A**, Sequence alignment of the C-terminal tails of GPRC5A from different species. **B**, The constructs of GPRC5A mutants used in this study. **C**,**D** *,* **E**,**F**, HEK293T cells were co-transfected with plasmids encoding EGFR or vector plus either GPRC5A-wild type (WT) or Y317/320 F or Y347/350 F or 4 F mutants as indicated. Cells were treated with or without EGF as indicated. Cell lysates were harvested for Western blots using the antibodies as indicated. **G**, HEK293T cells were co-transfected with the plasmids containing EGFR plus GPRC5A-WT or a series of GPRC5A mutants as indicated. Cellular proteins were analyzed by Western blot using antibodies as indicated.

To examine the relationship between EGFR and GPRC5A, we first co-expressed EGFR or vector with myc-tagged GPRC5A in HEK293T cells. Tyrosine phosphorylation of GPRC5A, identified using the PY99 antibody, was significantly increased in cells expressing EGFR. This phosphorylation was detected at 5 minutes after EGF treatment and reached to maximum levels in 6 hr (left panel, Figure 1A). However, in the absence of EGFR expression, no tyrosine-phosphorylation of GPRC5A was detected even after 6 hr EGF treatment (right panel, Figure 1A). Thus, the EGF-induced tyrosine phosphorylation of GPRC5A is mediated through EGFR. To investigate whether GPRC5A is a direct target of EGFR tyrosine kinase, we co-expressed EGFR and myc-tagged GPRC5A in HEK293T cells. After immunoprecipitating EGFR, we detected the associated GPRC5A in the presence or absence of EGF (left panel, Figure 1B), and vice versa (left panel, Figure 1C). In addition, GPRC5A was heavily tyrosine-phosphorylated when cells were treated with EGF (right panel, Figure 1B and 1C); however, this effect could be inhibited by tyrosine kinase inhibitor AG1478. Thus, our results indicate that: (1) the interaction of EGFR with GPRC5A is independent of EGF and the kinase activity of EGFR (left panel, Figure 1B); (2) activation of EGFR correlates with the level of EGF-mediated tyrosine phosphorylation on GPRC5A; and (3) EGFR inhibitor suppresses EGF-mediated GPRC5A phosphorylation.

### Y317 and Y320 at the C-terminal tail of GPRC5A are phosphorylated by EGFR

GPRC5A contains seven transmembrane regions and a C-terminal cytoplasmic tail. When the primary sequences of GPRC5A from difference species were aligned, we found that there are two potential double-tyrosine modules, including Y317/Y320 and Y347/Y350, located at the C-terminal tail of GPRC5A. These two double-tyrosine modules are highly conserved among all species (Figure 2A). To determine whether the predicted four tyrosine residues are responsible for EGF-induced tyrosine phosphorylation of GPRC5A, we constructed a GPRC5A-4 F mutant, in which four tyrosine residues (Y317, Y320, Y347 and Y350 site) were replaced by phenylalanine (F), that is unable to be phosphorylated (Figure 2B). When EGFR was co-expressed with GPRC5A-WT (wild type), we found that EGF induced tyrosine-phosphorylation of GPRC5A-WT (Figure 2C). However, when EGFR was co-expressed with 4 F mutant, no tyrosine- phosphorylation was induced by EGFR in GPRC5A-4 F (Figure 2D). Thus, it is likely that Y317, Y320, Y347 and Y350 are the major residues involved in the phosphorylation of GPRC5A mediated by EGFR.

To further determine which double-tyrosine modules were the major residues responsible for GPRC5A phosphorylation, we generated two additional GPRC5A mutants, Y317/320 F, and Y347/350 F (Figure 2B). When these two mutants were co-expressed with EGFR in HEK293T cells, we found that both Y317/320 F and Y347/Y350F were tyrosine-phosphorylated. However, the tyrosine-phosphorylation of Y317F/Y320F mutant is reduced more than that of Y347/350 F mutant (Figure 2E). The tyrosine phosphorylation was specifically mediated by EGFR since no tyrosine-phosphorylation was induced in absence of EGFR (Figure 2 F). These results suggest that Y317/Y320 are the preferred residues responsible for EGFR-mediated phosphorylation. In addition, we generated four individual tyrosine mutants (Y317F, Y320F, Y347F and Y350F) of GPRC5A (Figure 2B) and co-expressed them with EGFR in HEK293T cells. We found that Y317F and Y320F mutants exhibited lower tyrosine phosphorylation than that of Y347F and Y350F mutants (Figure 2G). This result is consistent with the observation that the Y317F/Y320F mutant had lower phosphorylation than that of the Y347F/Y350F (Figure 2E). Taken together, these results indicate that Y317 and Y320 are the preferred residues responsible for EGFR-mediated GPRC5A phosphorylation.

### Endogenous EGFR phosphorylates endogenous GPRC5A in NSCLCs

**Figure 3:**
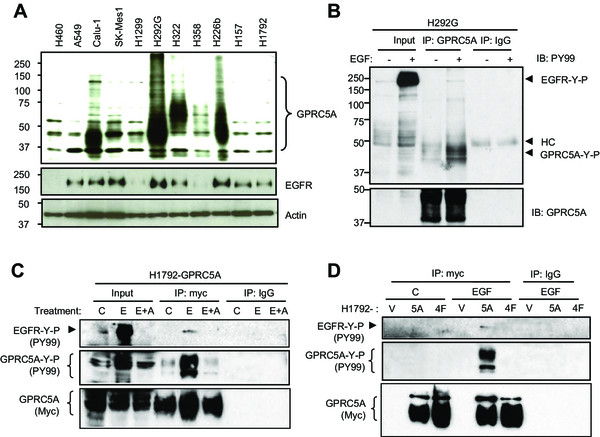
**Endogenous EGFR in NSCLC cells induces tyrosine phosphorylation on GPRC5A. A**, Western blot of eleven NSCLC cell lines was performed with anti-GPRC5A, anti-EGFR, anti-HER-2, and anti-actin, as indicated. **B**, H292G Cells were serum-starved for 24 hours, then treated with or without EGF (100 ng/ml) for 5 min. Cell lysates were harvested for IP-Western using either anti-GPRC5A or normal IgG. Western blot was performed using either PY99 antibody or anti-GPRC5A as indicated. **C.** H1792-GPRC5A, **D**, H1792-V, H1792-5A, H1792-4 F stable transfectants were serum-starved for 24 hours and then treated with EGF for 5 minutes or pretreated with AG1478 (30 ng/ml) for 6 hours as indicated. Total cellular proteins were analyzed by IP-Western using the antibodies as indicated.

Next, we investigated whether endogenous EGFR can phosphorylate endogenous GPRC5A in NSCLC cell lines. We examined 11 NSCLC cell lines for expression of GPRC5A, EGFR and HER2, and found that 4 out of 11 NSCLC cell lines (H292G, Calu-1, H322, and H226b) expressed elevated levels of GPRC5A (Figure 3A). Notably, all of these four cell lines also have relatively high levels of EGFR. This suggests that the endogenous RTKs of EGFR family members are available in these cells for providing potential tyrosine-phosphorylation on GPRC5A. After serum starvation for 24 hours, we treated H292G cells with EGF (100 ng/ml) in serum-free medium, then immunoprecipitated endogenous GPRC5A for immunobloting. The IP-Western assay showed that GPRC5A was indeed tyrosine-phosphorylated after EGF exposure (Figure 3B), indicating that endogenous EGFR can interact and phosphorylate GPRC5A.

To expand the characterization of the tyrosine-phosphorylation of GPRC5A, we developed GPRC5A-WT (5A) and GPRC5A-4 F (4 F) stable transfectants in H1792. NSCLC cell line H1792 was selected for two reasons: 1) H1792 cells express a low level of endogenous GPRC5A (Figure 3A), thus expression of exogenous GPRC5A-WT and GPRC5A-4 F could be detected and evaluated; and 2) this cell line expresses endogenous EGFR (Figure 3A), thus EGFR activity can be induced by EGF. To determine the effects of endogenous EGFR on GPRC5A phosphorylation, we treated GPRC5A-WT stable H1792 transfectants (H1792-GPRC5A) with medium (C), EGF (E), or EGF plus pretreatment with EGFR inhibitor AG1478 (30 ng/ml for 6 hours) (E + A). We found that, GPRC5A was tyrosine-phosphorylated after EGF treatment, and this phosphorylation was blocked when cells were treated with AG1478 (Figure 3C).

Next, we determined whether endogenous EGFR-mediated tyrosine phosphorylation occurs at the four identified tyrosine residues in GPRC5A. Using the H1792 stably-expressing cell lines (V, 5A, and 4 F) in the presence or absence of EGF (100 ng/ml, 5 minute treatment), we found that the tyrosine phosphorylation of GPRC5A-WT (5A) was significantly induced by EGF in H1792-GPRC5A (5A) cells (Figure 3D). However, no tyrosine phosphorylation was found in H1792 cells expressing vector (V) or GPRC5A-4 F (4F). The lack of phosphorylation in H1792-4 F cells was not due to the lack of GPRC5A-4 F expression, as similar levels of GPRC5A were immunoprecipitated from H1792-5A and H1792-4 F cells. Together, these results indicate that endogenous EGFR can phosphorylate GPRC5A at four identified tyrosine residues (Y317, Y320, Y347 and Y350) in response to EGF stimulation in the lung cancer H1792 cells.

### Tyrosine phosphorylation inhibits the tumor suppressive activities of GPRC5A

**Figure 4:**
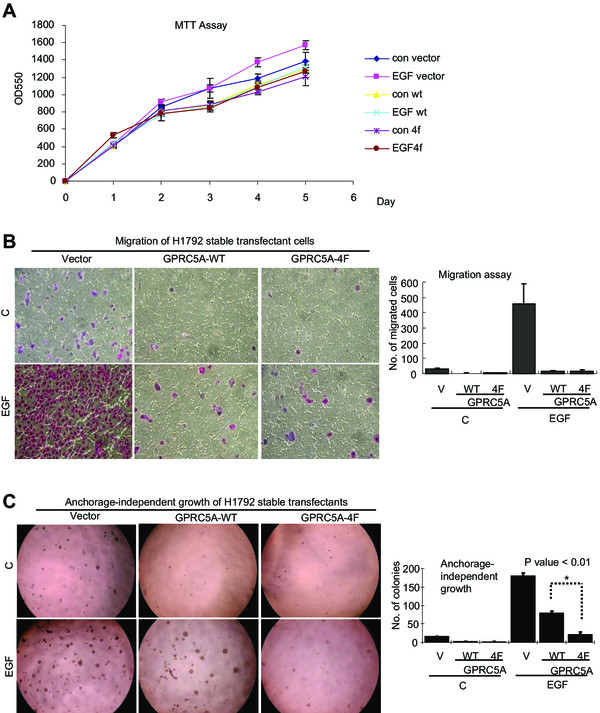
**GPRC5A-mediated inhibition of cell invasion and anchorage-independent growth of NSCLCs was repressed by tyrosine phosphorylation. A**, The proliferation rate of H1792 cells stable transfected with vector, GPRC5A-WT, or -4 F were grown in 96-well plates in growth medium with 10% FBS with or without 100 ng/ml EGF for 24, 48, 72 , 96 and 120 hours. The number of cells in each well was measured by the MTT assay. **B**, The migration of H1792 stable transfectants as indicated was assayed. The photomicrographs of cells on the bottom side of the filter (migrated cells) (*left*) and the mean (and 95% confidence intervals) number of migrated cells was shown in the bar graph (*right*). The differences between the number of cells in EGF-treated GPRC5A-WT and -4 F stable transfectants in H1792 cells were not statistically significant (P >0.05; two-sided z test). **C**, anchorage-independent growth was assessed in GPRC5A-WT and -4 F transfected H1792 cells. The cells were resuspended in agarose/Matrigel and analyzed for colony formation over 2 weeks. The photomicrography of colonies (*left*), and the number of colonies (bar on *right*) were shown. The differences between the number of colonies in EGF-treated GPRC5A-WT and 4 F mutant H1792 cells were statistically significant (P <0.01; two-sided z test).

To determine the biological effects of tyrosine phosphorylation on GPRC5A, we examined cell growth, migration, and anchorage-independent growth in H1792 cells stably expressing GPRC5A-WT (H1792-5A) or GPRC5A-4 F (H1792-4 F). We found no significant difference in cell proliferation among H1792-5A and H1792-4 F cells both before and after EGF treatment (Figure 4A). Noticeably, EGF treatment significantly increased the number of migrated H1792-V cells compared to the untreated cells (Figure 4B). We found that EGFR-mediated cell migration was inhibited by overexpression of GPRC5A-WT and 4 F (Figure 4B). However, the differences between the number of migratory cells in EGF-treated GPRC5A-WT and -4 F stable transfectants in H1792 cells were not statistically significant (P > .005; two-sided z test) (Figure 4B). This result suggests that EGF-mediated tyrosine phosphorylation of GPRC5A does not affect the suppressive effect of GPRC5A on cell migration.

We then examined the anchorage-independent growth in these cells before or after EGF stimulation. We found that expression of either GPRC5A-WT or GPRC5A-4 F inhibited colony formation in H1792 cells compared with that of vector control in absence of EGF treatment (top panel, Figure 4C). EGF treatment significantly increased the colony formation both H1792-V and H1792-5A cells, however no EGF-effect was found in H1792-4 F cells (bottom panel, Figure 4C). This indicates that EGF-induced tyrosine phosphorylation inhibited the suppressive effect of GPRC5A but not the 4 F mutant on anchorage-independent growth. Taken together, these results demonstrate that EGF-induced tyrosine phosphorylation on GPRC5A inactivates the suppressive activities of GPRC5A on anchorage-independent growth in H1792 cells.

### GPRC5A exists as a non-phosphorylated form in normal lung tissue and tyrosine-phosphorylated form in NSCLC tissues

**Figure 5:**
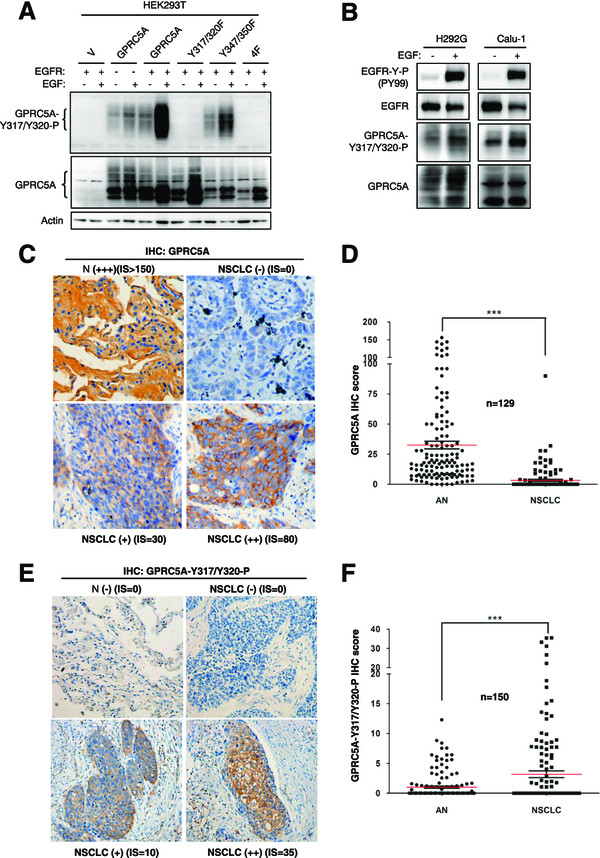
**The tyrosine-phosphorylated GPRC5A expression is increased in NSCLC histological tissue specimens. A**, HEK293T cells were co-transfected with plasmids of EGFR, GPRC5A WT, and mutants, as indicated. Two days later, the cells were treated with or without EGF. Cell lysates were harvest for Immunoblot using anti-tyrosine-phosphorylated GPRC5A (Y317-P/Y320-P) and anti-GPRC5A. **B**, H292G and Calu-1 cells were pretreated with DMEM containing 0% overnight, then treated with or without EGF (50 ng/ml) for 3 hour as indicated. Cell lysates were harvested for Immunoblot using anti-EGFR-P (Y1068), anti-EGFR, anti-tyrosine-phosphorylated GPRC5A or anti-GPRC5A as indicated. The representative photomicrographs of IHC staining were shown for normal lung and NSCLC tissues with ant-GPRC5A (n = 129) **(C)** and anti-tyrosine-phosphorylated GPRC5A (n = 150) **(E)**. Negative (-), positive (+), (++), (+++) staining samples, and the IHC score (IS), were as indicated. Dot-plots depicting GPRC5A **(D)** and the tyrosine-phosphorylated **(F)** expression in IHC staining were shown for adjacent normal lung (AN) and NSCLC samples. The differences between normal lung and NSCLCs were statistically significant as indicated as *(P <0.01; two-sided z test).

To determine the phosphorylated status of endogenous GPRC5A in normal lung and lung tumor tissues *in vivo*, we developed specific antibody against the double-tyrosine phosphorylation (Y317/Y320) sites of GPRC5A. Using co-transfection and immunoblot assay, we found that this antibody detected EGFR-mediated tyrosine phosphorylation of GPRC5A and Y347/350-F, but not Y317/320-F and 4 F (Figure 5A). Thus, the antibody is specifically for Y317/Y320 phosphorylated sites of GPRC5A (Figure 5A).

Next, we examined, via this antibody, the phosphorylation status of GPRC5A in H292G and Calu-1 cells which express endogenous GPRC5A. Immunoblot shows that EGF treatment (100 ng/ml for 4 hours) increased the level of the phosphorylated GPRC5A (Figure 5B); Taken together, these results indicate that EGF treatment also increased phosphorylation of Y317/Y320 sites in endogenous GPRC5A.

To determine the phosphorylation status of GPRC5A in normal lung and lung tumor tissues, we performed immunohistochemical (IHC) staining analysis by using antibodies to either GPRC5A or tyrosine-phosphorylated-GPRC5A (Y317/Y320-P) in 129 or 150 paired adjacent normal lung tissues and NSCLC tissues, respectively. The results of IHC staining show that total GPRC5A expression was significantly higher in adjacent normal lung tissues than NSCLC ones (Figure 5C-D), which is consistent with the previous report ( [2, 5]), supporting GPRC5A is a lung tumor suppressor. Interestingly, the tyrosine-phosphorylated GPRC5A, although the total level was low, was significantly higher in NSCLC tissues than normal lung tissues (Figure 5E-F). These results suggest that, in normal lung tissue, GPRC5A was non-phosphorylated; whereas in lung tumor tissues, GPRC5A became highly tyrosine-phosphorylated, supporting that GPRC5A in lung tumor tissues are the function-defective ones.

## Discussion

In this study, we showed that EGFR interacts with and phosphorylates GPRC5A, leading to inactivation of the tumor suppressive function of GPRC5A in lung cancer cells. In this study, EGFR and GPRC5A were found to form a complex together. It remains unclear whether this interaction is directly or indirectly and whether other adaptor molecules, such as Grb-2, are involved in the interaction of EGFR-GPRC5A complex. Further investigation will reveal detailed mechanistic insight for this interaction.

Using systematic site-directed mutagenesis analysis, we identified that Y317, Y320, Y347 and Y350 are involved in the tyrosine phosphorylation of GPRC5A. In a whole-cell proteomic phospho-peptide analysis, Y317, Y320, Y347 and Y350 of GPRC5A were found to be phosphorylated in cells overexpressing EGFR and Src by mass Spectrometry [22, 23]. These results support our finding that the two double-tyrosine modules in GPRC5A are the major residues responsible for tyrosine phosphorylation. Interestingly, Y317F exhibited much reduced tyrosine phosphorylation than Y320F. One possible explanation is that Y317 is a primary/initial site for tyrosine phosphorylation, whereas Y320 is phosphorylated subsequently. This interesting phenomenon is analogous to GSK-3β-mediated β-catenin phosphorylation, in which CK1 induced-phosphorylation on Ser45 of β-catenin facilitates/primes GSK-3β-mediated sequential phosphorylation on Thr41, Ser37, and Ser33. This reverse order of phosphorylation results in the degradation of phosphorylated β-catenin by ubiquitin–proteasome system [24]. It is likely that phosphorylation of one residue leads to conformation change of the protein, which in turn results in exposure of other target residue for subsequent phosphorylation. In addition, different phosphorylation motif may have different biochemical or biological changes. For example, we previously demonstrated that two phosphorylation motifs on Snail regulate two different functional properties of Snail, one controls the subcellular localization whereas the other controls proteins ubiquitination and degradation of snail [25]. In this study, we found that the major phosphorylation sites are in the first double-tyrosine module (Y317 and Y320) in the C-terminal domain of GPRC5A. It is unclear whether other tyrosine kinases are responsible for the phosphorylation of the second double-tyrosine module (Y347 and Y350) of GPRC5A.

Several reports suggest that GPRC5A could be tyrosine phosphorylated in the C-terminal domain, and different stimuli in different cell lines or tissues may induce different tyrosine phosphorylation patterns. For example, HRG-treated 184A1 HMECs cells, which overexpress HER-230-fold, showed 332 tyrosine-phosphorylation sites in 175 proteins. Interestingly, Y347 on GPRC5A (RAI3) was among these peptides [23]. These discrepancies between theirs and our studies may be due to different tissues or experiment conditions. It is also possible that Y347/Y350 might be preferentially phosphorylated by other tyrosine kinases in different cellular context.

The biological activities of GPRC5A appeared to be regulated differently by tyrosine phosphorylation. First, GPRC5A did not affect cell proliferation, regardless of tyrosine phosphorylation status. Second, GPRC5A-mediated inhibition of cell migration is independent of tyrosine phosphorylation. These results are consistent with Kumar’s finding, in which tyrosine phosphorylation of GPRC5A showed no effect on cell proliferation and migration ability [26]. And third, tyrosine phosphorylation did decrease or abolish GPRC5A-mediated inhibition on EGF-enhanced anchorage-independent growth of H1792 cells. Taken together, these observations strongly support out hypothesis, that EGFR-mediated tyrosine phosphorylation of GPRC5A inactivates some of the tumor suppressor activities of GPRC5A. Thus, targeting EGFR by RTK inhibitors will restore the tumor suppressor functions of GPRC5A in lung cancer cells.

Importantly, IHC analysis showed that GPRC5A in adjacent normal lung tissues is non-tyrosine- phosphorylated, whereas it is tyrosine-phosphorylated in NSCLCs. This observation strongly supports the model that tyrosine phosphorylation inhibited the tumor suppressive activities of GPRC5A. Because GPRC5A in H292G cells was tyrosine-phosphorylated either with or without EGF *in vitro*, we assume that GPRC5A could be either tyrosine-phosphorylated by EGFR or other receptor tyrosine kinases (RTKs). Thus, we proposed that the tyrosine-phosphorylated GPRC5A could be used as a prognostic marker for tumor progression. Thus, targeting receptor tyrosine kinases will be an effective in preventing and treating lung cancer by restoring the tumor suppressor function of GPRC5A. Our results provide a novel mechanism in supporting this strategy.

## Materials and methods

### Cell culture and reagents

Human NSCLC cell lines (H460, A549, H226, H157, Calu-1, H226B, H322, H292G, H1792, H358 and Sk-Mes-1) were obtained from Adi Gazdar and John Minna (UT Southwestern, Dallas, TX). Cell culture was performed as described previously [2]. EGF was obtained from R&D system (Minneapolis, MN, USA). AG1478 was purchased from Merck Millipore (Billerica, MA, USA). EGFR mouse mAb (#2256, IP), EGFR Rabbit mAb (#4267), were from Cell Signaling Technology. Antibody to P-Tyr (PY99) was from Santa Cruz Biotechnology. Antibodies against myc and actin were from Sigma-Aldrich. HRP-conjugated secondary antibodies were from eBioScience (San Diego, CA). Rabbit polyclonal antibody to human GPRC5A was as described previously [2]. Mouse monoclonal antibody (clone 1 L23) to the C-terminus of GPRC5A (PYKDYEVKKE) was developed and purified in Abmart (Shanghai, China) [27]. Polyclonal rabbit anti-GPRC5A-Y317/Y320-P antibody was developed against DTLYpAPYpSTH from Abmart (Shanghai, China).

### Plasmids and transfection assay

HEK293T cells were transfected using Lipofectamine 2000 transfection reagent (Invitrogen, Grand Island, NY). Transfected plasmids include: pcDNA-EGFR, pcDNA-GPRC5A-myc, and GPRC5A mutants. Cells were starved in serum-free medium for 24 hours, then treated with EGF (100 ng/ml) for various time periods as indicated.

### Construction of wild-type and mutant GPRC5A plasmids

Plasmid pcDNA3.1 (+)-GPRC5A-Myc was as described [2]. GPRC5A mutants were constructed with the following primers: Primer Y317/320 F-F: (5′-GGT TTT GAA GAG ACC GGT GAC ACG CTC TTT GCC CCC TTT TCC ACA CAT TTT C-3′) and primer Y317/320 F-R (5′-GAA AAT GTG TGG AAA AGG GGG CAA AGA GCG TGT CAC CGG TCT CTT CAA AAC C-3′). Primer Y347/350 F-F: (5′-CCA CGC TTG GCC GAG CCC TTT TAA AGA CTT TGA AGT AAA GAA AGA GG-3′) and primer Y347/350 F-R: (5′-CCT CTT TCT TTA CTT CAA AGT CTT TAA AAG GGC TCG GCC AAG CGT GG-3′). 4 F mutant (GPRC5A-Y317/320/347/350 F-myc) of GPRC5A was generated by ligation of GPRC5A-Y317/320 F-myc and GPRC5A-Y347/350 F-myc after digestion with KpnI and EcoRI separately. All sequences were verified by DNA sequencing.

### Transient and stable transfection of cells

Transfection of H1792 cells with plasmids was performed by electrophoresis: Briefly, the cells were harvested, and about 3 × 10^5^ cells were mixed with 100 μl of electroporation transfection solution (Solution V, Dharmacon, Lafayette, CO), plus plasmids, and the mixtures were transferred to electroporation cuvettes and subjected to electroporation (Amaxa Biosystems, Cologne, Germany) according to the manufacturer’s pro-grams and instructions.

### Immunoprecipitation (IP) and western blotting

IP was performed with 2 μg of antibodies against myc or EGFR or normal IgG (N IgG) (as a negative control) in 1.0 mg whole cell lysate. Immunoblot [25, 28] and cell fractionation [29] was performed as described.

### Cell migration assay

The trans-well cell migration system consisted of cell culture inserts with an 8.0 μM pore size in 24-well plate (BD BioCoat #354578, San Jose, CA). Cells were resuspended with fetal bovine serum (FBS)-free DMEM, and seeded onto the insert (4 × 10^4^ cells). DMEM (10% FBS) with or without EGF (100 ng/ml) was loaded into the lower chamber and incubated overnight. Migrated cells, which attached to the lower side of the filter were fixed with 96% ethanol for 30 minutes and stained with 1.5% crystal violet. Migrated cells were counted in five different microscopic fields by 10 × magnifications using a Nikon fluorescence microscope.

### Anchorage-independent colony formation in soft agar assay

Soft-agarose assay was performed as described previously [2]. Aggregates of 50 or more cells were considered to be a colony. Colonies were counted in four different fields under a microscope at 4× magnification and photographed. The means and 95% confidence intervals (CIs) of the number of colonies in four microscopic fields were calculated. Two independent experiments in triplicates were performed.

### Tissue samples

We obtained archival, formalin-fixed and paraffin-embedded (FFPE) material from surgically resected lung cancer specimens containing tumor and paired adjacent non-malignant epithelium tissue from the Shanghai Chest Hospital from 2008 to 2013 (Shanghai, CHINA). The adjacent non-malignant epithelia were collected at sites at least 2 cm away from the edge of tumor mass, with best efforts of avoiding contamination by the tumor cells. In total, 150 primary NSCLC patients without prior radiotherapy or chemotherapy were enrolled in this study. All NSCLC samples were confirmed histologically, and tumor samples were rechecked to ensure that tumor tissue was present in more than 80% of the specimens.

### Immunohistochemistry

The samples of lung tumor and adjacent normal lung tissues were fixed with formalin buffer and embedded in paraffin. Immunohistochemical (IHC) staining was performed on 3-μm sections of paraffin-embedded specimens with the use of mouse monoclonal antibody (clone 1 L23) to the C-terminus of GPRC5A (PYKDYEVKKE) or rabbit anti-GPRC5A-Y317/Y320-P. These antibodies were developed and purified in Abmart (Shanghai, China). The process of IHC was performed as previously described [27].

### Statistical analyses

All analyses were performed in triplicates, and the significance of differences between groups was calculated using the Student’s t test. P values <0.05 were considered to be statistically significant.
